# Where was this thing again? Evaluating methods to indicate remembered object positions in virtual reality

**DOI:** 10.1167/jov.24.7.10

**Published:** 2024-07-12

**Authors:** Immo Schuetz, Bianca R. Baltaretu, Katja Fiehler

**Affiliations:** 1 Experimental Psychology, Justus Liebig University, Giessen, Germany; 2 Center for Mind, Brain and Behavior (CMBB), Philipps University Marburg and Justus Liebig University, Giessen, Germany

**Keywords:** object placement, pointing, virtual reality, memory-guided action, accuracy, precision

## Abstract

A current focus in sensorimotor research is the study of human perception and action in increasingly naturalistic tasks and visual environments. This is further enabled by the recent commercial success of virtual reality (VR) technology, which allows for highly realistic but well-controlled three-dimensional (3D) scenes. VR enables a multitude of different ways to interact with virtual objects, but only rarely are such interaction techniques evaluated and compared before being selected for a sensorimotor experiment. Here, we compare different response techniques for a memory-guided action task, in which participants indicated the position of a previously seen 3D object in a VR scene: *pointing*, using a virtual laser pointer of short or unlimited length, and *placing*, either the target object itself or a generic reference cube. Response techniques differed in availability of 3D object cues and requirement to physically move to the remembered object position by walking. Object placement was the most accurate but slowest due to repeated repositioning. When placing objects, participants tended to match the original object's orientation. In contrast, the laser pointer was fastest but least accurate, with the short pointer showing a good speed–accuracy compromise. Our findings can help researchers in selecting appropriate methods when studying naturalistic visuomotor behavior in virtual environments.

## Introduction

Interacting with everyday objects in our environment requires a number of complex perceptual, cognitive, and motor processes. Think of grasping a coffee-filled cup and taking a sip: Understanding how humans perform this simple everyday action requires perceiving the environment, identifying the target objects therein and their spatial configuration, planning an action such as grasping, and then performing the grasp movement based on the target's current or remembered location. In recent investigations, the focus of sensorimotor research has branched out from abstract and highly controlled laboratory experiments toward more complex, naturalistic environments and tasks (Fiehler & Karimpur, 2023). For this latest research area, virtual reality (VR) technology allows for the immersion of participants in high-realism, naturalistic environments, which are nevertheless controllable by the experimenter to great detail (Fox, Arena, & Bailenson, 2009; Scarfe & Glennerster, 2015). This provides the opportunity to increase availability and external validity of stimuli in human behavioral research, such as spatial perception and memory tasks (Draschkow, 2022; Rivu et al., 2021). VR further facilitates a multitude of interactions with virtual content and objects (Bergström, Dalsgaard, Alexander, & Hornbæk, 2021; LaViola, Kruijff, McMahan, Bowman, & Poupyrev, 2017) and allows for large-scale virtual scenes that enable participants to walk around and explore content. At the same time, natural object interactions such as grasping are currently still limited compared to real-world lab setups due to missing haptic feedback and temporal tracking delays (e.g., Chessa, Maiello, Klein, Paulun, & Solari, 2019; Fiehler & Karimpur, 2023). Due to the growing commercial success of VR devices such as the HTC Vive/Vive Pro Eye and Meta (previously Oculus) Quest series head-mounted displays (HMDs), VR is nevertheless seeing increased adoption in research labs.

Recent studies have utilized VR to study scene perception and spatial cognition (Brookes, Warburton, Alghadier, Mon-Williams, & Mushtaq, 2019; Draschkow & Võ, 2017; Karimpur, Kurz, & Fiehler, 2020; Klinghammer, Schütz, Blohm, & Fiehler, 2016) using a diverse set of interaction paradigms. For example, one study instructed participants to arrange a set of related objects in a virtual room using a VR controller and investigated how semantic rules influenced their performance in a subsequent memory test (Draschkow & Võ, 2017). Another paradigm included a test of participants’ spatial memory by presenting all previously seen objects in a random pile and requiring participants to place all objects in their remembered position (Helbing, Draschkow, & Võ, 2020). Other studies instructed participants to point to the remembered location of target objects using their tracked index finger in VR (Karimpur, Eftekharifar, Troje, & Fiehler, 2020; Karimpur, Morgenstern, & Fiehler, 2019; Klinghammer et al., 2016) or by placing a tracked physical object (Karimpur, Kurz, et al., 2020). Finally, another study applied an interaction method resembling the classic “flashlight” pointing mechanism (Liang & Green, 1994), instructing participants to use a controller-based flashlight to highlight the target object during visual search in a dark room (Beitner, Helbing, Draschkow, & Võ, 2021). Experiments such as these display the possibilities for interaction in sensorimotor VR studies.

In the field of human–computer interaction (HCI), basic object-related interactions such as positioning, rotating, and scaling have been intensively studied and are now well understood (Argelaguet & Andujar, 2013; Dang, 2007; LaViola et al., 2017). One of the most frequently used interaction classes is *pointing* (for reviews, see Argelaguet & Andujar, 2013; Dang, 2007), often implemented using a “raycast” mechanic in which a virtual ray extends from the user's hand or controller, and the intersection point between the ray and the virtual scene is visualized, for example, using a line or cursor (Mine, 1995). Another class of interactions concerns object manipulation or *placement*, which includes transformations such as position, rotation, and scale and can be controlled using a variety of input devices from controllers to hand input (Mendes, Caputo, Giachetti, Ferreira, & Jorge, 2019). Accordingly, a large body of HCI research aims to model human performance in pointing and “selection” tasks (typically achieved by pointing at one out of multiple objects using online visual feedback and confirming selection using a button; Grossman & Balakrishnan, 2004; Wingrave & Bowman, 2005). For example, one study compared pointing with fixed and unlimited ray pointers in VR and reported faster responses and fewer errors with the unlimited pointer but higher response throughput with the fixed pointer length (Batmaz & Stuerzlinger, 2020). Other examples include moving and rotating objects to a specified target position (Mendes et al., 2019) or modeling interaction efficiency using the well-known Fitts's law (Fitts, 1954; MacKenzie, 1992) when selecting targets in three-dimensional (3D) space (Grossman & Balakrishnan, 2004; Wingrave & Bowman, 2005). A review article by Bergström et al. (2021) summarizes two decades of work in 3D object selection and manipulation, with the goal of determining guidelines for fair evaluation and comparison of interaction paradigms in VR.

The works described above mainly focused on online visuomotor actions, where a target object is continuously visible and/or the goal position to which an object needs to be transported is clearly indicated. However, everyday tasks commonly include memory-guided actions, in which the object and/or goal position is not currently visible but must be retrieved from memory—for example, when pointing to where an object was previously located or when reaching for the aforementioned cup of coffee while reading an email. The present study aimed to investigate whether the behavior reported for online visuomotor actions also transfers to memory-guided actions in VR. Our paradigm was inspired by recent work on visual memory and spatial cognition (Draschkow & Võ, 2017; Helbing et al., 2020; Karimpur et al., 2019; Karimpur, Eftekharifar, et al., 2020; Karimpur, Kurz, et al., 2020; Klinghammer et al., 2016), in which participants were asked to reproduce the position of a previously seen object by manual pointing or placing the object in its previous location. Their accuracy in doing so, defined as the distance to the original object position, was then used to measure spatial memory performance and how it might be influenced by different visual and scene-related factors. Interaction methods in these VR experiments were selected based on task and study constraints, such as using manual pointing for an experiment where targets were always on a table in front of the participant (e.g., Klinghammer et al., 2016). However, so far, no study has directly compared the performance of different methods to indicate a remembered 3D position in VR, which would allow researchers to choose an interaction technique that directly optimizes for either spatial response accuracy or experiment duration (i.e., number of responses per session).

In the present study, we systematically compared four different response techniques using a handheld VR controller, separated into two major classes of response: *placement* and *pointing*. In the *object placement* technique, participants were asked to place the target object accurately in the previously seen location (cf. Draschkow & Võ, 2017; Helbing et al., 2020). In the *cube placement* technique, they could place a generic cube at the remembered target location. The cube could be moved and rotated in the same way as described above but provided no information about the target object's size, center of mass, or even its identity, which might be a requirement in a spatial memory study. We further investigated two pointing techniques, in which participants used a fixed- or unlimited-length laser pointer (cf. Batmaz & Stuerzlinger, 2020) to indicate remembered object positions. In the *fixed pointing* technique, participants were required to physically move to the target object's location within the scene due to the short pointer length. In contrast, the *laser pointing* technique behaved like a physical “laser pointer,” meaning that participants could point to any location on a surface from a distance.

For each of the four response techniques, we measured *spatial accuracy* and *precision* of the indicated remembered object position. For the placement techniques, we additionally considered *rotational accuracy* since objects could be both translated and rotated in 3D space. We further measured *response duration* as the time between when participants first saw the scene without the target object and when they confirmed their final response via a button press. Finally, we were interested in how subjectively easy or hard participants perceived each of the techniques by measuring *subjective workload* for each technique using the NASA task-load index (TLX) questionnaire (Hart, 2006).

## Methods

### Participants

Sixteen volunteers participated in the study (age 19–35 years, mean 27.4 ± 4.6 years; 12 female, 4 male). All participants had normal or corrected-to-normal vision, wore appropriate vision correction where necessary (three contact lenses, four glasses), and were right-handed as confirmed using the Edinburgh Handedness Inventory (EHI; Oldfield, 1971, defined as EHI value greater than zero). Participants gave written informed consent before the start of the experiment and received no compensation for their participation. The experiment was approved by the local ethics committee and followed the Declaration of Helsinki (2008).

### Experimental setup and scenes

The study took place in a 6.7-m × 4.9-m lab room equipped with four SteamVR 2.0 base stations for positional tracking (“lighthouses”; Valve Corp., Bellevue, WA, USA), one mounted in each corner. Participants wore an HTC Vive Pro Eye HMD (HTC Corp., Xindian, New Taipei, Taiwan) and held an HTC Vive Pro controller (HTC Corp.) in their right hand. We selected this controller for the study due to its widespread availability and common usage in VR interaction studies (Bergström et al., 2021). The experiment was implemented in Unity (Version 2020.3; Unity Technologies, Inc., San Francisco, CA, USA), SteamVR (Version 1.20.1), and the Unity Experiment Framework (Brookes et al., 2019). It was run on a Dell Alienware desktop PC (Intel Core i9 CPU, 2.6 GHz, 32 GB RAM, Dual NVidia GeForce GTX1080 Ti GPU).

**Figure 1.**
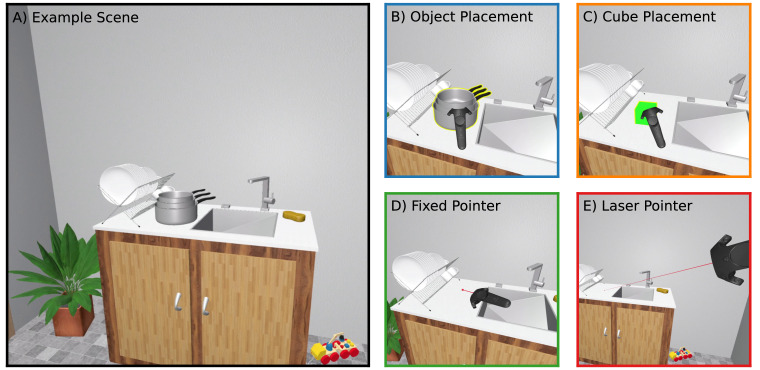
Example virtual scene (**A**: kitchen environment used in practice trials) together with the four response techniques (**B**–**E**) used to indicate remembered object position in this study: placing a copy of the target object using the controller (**B**), placing a reference object (**C**), pointing with a fixed-length pointer (**D**), and pointing far using a virtual laser pointer (**E**). The stack of pots in (**A**) was the target object in this example. Border colors identify the same techniques in later figures.

**Figure 2.**
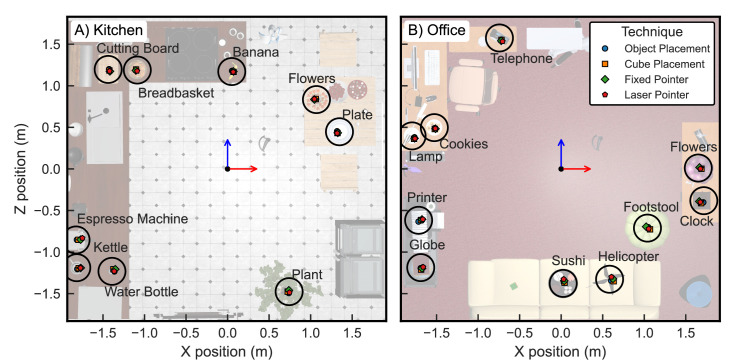
Top-down view of the two virtual scenes used (**A**: kitchen, **B**: office), with plot axes indicating Unity coordinates in meters. Target objects whose position needed to be reported are marked with black circles and labels. Average response endpoints across all participants are shown as colored markers for each response technique.

During the experiment, each participant was presented with a virtual indoor scene of either a *kitchen* or *office* environment (pseudo-randomly assigned to participants; cf. Figure 1A). Each virtual room measured approximately 3.8 m × 3.5 m and thus fit completely within the lab room, allowing participants to move around the scene by walking, instead of implementing a “teleportation” method to move around the scene. The scenes were previously designed for spatial memory paradigms, and each contained 35 different objects including furniture, decorations, and scene-appropriate everyday objects (e.g., pots in the kitchen scene in Figure 1; for details, see David, Beitner, & Võ, 2021; Helbing et al., 2020). We selected 10 objects in each scene as *target objects* but had to exclude one object (a painting in the kitchen scene) due to a data recording error. The full set of 19 target objects and their positions within each scene is displayed in Figure 2. Objects were arranged approximately radially around the central starting position at distances ranging from 1.2 to 3.6 m (average: 2.4 m; distances defined in the X–Z plane as depicted in Figure 2). The central area of the scene was free of virtual “obstacles” to allow unrestricted walking. All objects were depicted as resting on a surface; however, we fixed the initial object's position so that gravity did not affect objects in the virtual environment.

A third virtual scene with entirely different objects and layout than the two experimental scenes was used to familiarize participants with VR and practice the tasks before participating in the main experiment (see Figure 1A for an example view of the practice scene).

### Experimental paradigm

Participants performed four separate experimental sessions, each implementing one of the four techniques to report remembered object positions. Each session contained 50 individual trials (five presentations of 10 target objects) and took between 10 and 15 min. One half of participants performed the task in the kitchen scene, the other in the office scene (pseudo-randomly assigned). Initial analyses showed no difference between scenes for all metrics; therefore, all data were analyzed independent of scene. The entire experiment lasted about 1 hr. Before each trial, participants had to locate the starting position, indicated by a transparent blue pair of shoes displayed on the floor. They were required to stand on the starting position and face in the direction cued by the shoes, then press the trigger button on the controller to begin the trial. If a participant faced away from the cued direction by more than 40° or stood further away from the starting position than 30 cm, a buzzer sound was played and they had to correct their position before being able to continue. This approach ensured a consistent initial view of the scene for each trial. The upcoming target object was always indicated to the participant using floating text prior to starting each trial (e.g., “Pots” in the case of Figure 1A).

Each trial began with a *preview phase*, in which the entire scene (including the target object) was visible and could be freely explored by looking around until the participant pressed the trigger button. The scene was then replaced by a gray screen for 2 s, after which it reappeared but with the target object missing. The target object was always within the forward view based on a trial's starting location, meaning participants did not have to look behind themselves to find it. In the following *response phase*, participants were instructed to “report the remembered position of the target object” using one of the four different positioning techniques for each session:

• In the *object placement* technique, they were presented with the target object floating in front of them, which they could interact with using the controller (Figure 1B). A yellow outline was shown when the controller intersected the object, indicating that the object could be “grabbed” by holding the controller's side (“grip”) buttons and moved anywhere in the scene in any orientation. When the side buttons were released, the object stayed in place (i.e., was not affected by gravity). Participants could grab and release the object an unlimited number of times. Once the object was positioned at the desired location, they pressed the controller's trigger button to confirm its position and end the trial. Note that no specific instruction about object orientation was given.
• In the *cube placement* technique (Figure 1C), participants reported target object positions in the same way, but the object that was placed was always the same reference object, a green cube of 10 cm side length.
• In the *fixed pointer* technique (Figure 1D), a fixed-length (10 cm) red line was rendered attached to the virtual controller, which ended in a 1-cm diameter red sphere (“laser dot”). Here, participants were instructed to “move the red sphere to the remembered object position,” then press the trigger button to confirm and end the trial.
• Finally, in the *laser pointer* technique (Figure 1E), a similar but unlimited red line was emitted from the controller and the red sphere was always displayed at the point where a virtual raycast along this line intersected with an object or wall in the scene, mimicking a real laser pointer. Again, participants were instructed to press the trigger when done, confirming the pointing endpoint and ending the trial.

Due to the large number of participants required to fully counterbalance all four tasks (24 per scene = 48 participants), we instead ensured that each response technique appeared equally often across the four experimental sessions.

### Data recording

Pilot testing determined that participants primarily defined an object's position using the area that was in contact with the surface it rested on, for example, referencing the midpoint of the bottom flat surface of a vase of flowers as its position. We therefore defined true object positions as the 3D Cartesian coordinates (X, Y, Z) of the center of this bottom contact area (“contact points,” or “pivot points” in Unity parlance), measured in meters. When participants ended a trial, *response endpoints* were determined by the coordinates of the same contact point on the placed target object or cube (for the placement techniques) or by the center of the red sphere (“laser dot,” for fixed and unlimited pointing). *Positional errors* were defined as the 3D Euclidean distance between each endpoint and the corresponding true target position. We also computed a “surface error” measure that ignored differences in vertical position (i.e., X–Z plane as in Figure 2). This distance measure produced overall smaller numerical error values but yielded the same statistical effects as the 3D error metric; therefore, we chose to only report 3D position errors. *Orientation error* was defined as the angular difference between the original object's forward vector (i.e., the Z-axis of the object's local coordinate system in Unity) and the same vector of the placed target object or cube after the participant confirmed their response. To compute orientation error on each axis, we first computed the rotation matrix that would align both forward vectors for each trial, then extracted its yaw, pitch, and roll Euler angles. Finally, *response duration* was defined as the time between the onset of the response phase (i.e., when the scene reappeared with the target missing) and the time when participants ended the trial via controller button press, in seconds. For the pointing techniques, the button press immediately recorded the position of the red sphere as the participant's response, while in the placement techniques, they could grab and adjust the object or cube multiple times and pressed the trigger button to indicate trial completion. After each session (each presenting one response technique), participants filled out a NASA TLX questionnaire relating to their *subjective mental and physical effort* and task success in the preceding session (Hart, 2006). We subsequently analyzed the raw (unweighted) global TLX score as a metric of subjective workload for each technique. Finally, we recorded the 3D position and orientation of the headset and controller at 90 Hz (i.e., once per display frame) throughout each trial. In the object or cube placement techniques, we also recorded whether the object was currently grabbed or not and computed the number of object interactions per trial (i.e., how many times the object was grabbed and released using the controller).

### Data processing and analysis

Data analysis was performed using Python (Version 3.8) and R (Version 4.0.2; R Core Team, 2020). A total of 3,200 individual trials were recorded (16 participants × 4 techniques × 10 targets/scene × 5 responses/target = 3,200), but we removed 160 total trials due to a data recording error for one object, bringing the total number of responses to 3,040. Before analysis, we removed responses with 3D position errors (see below) of more than 2.5 standard deviations (*SD*) above the mean (≥19.4 cm) as outliers. This filter removed 32 trials (1.05%), and in almost all cases, the participant had accidentally pressed the trigger button before indicating the correct position, ending the trial early. We additionally filtered trials with response durations of 2.5 *SD* above the mean (≥14.3 s), which removed 76 trials (2.5%). These overly long trials were generally caused by participants asking questions or exploring the scene for a very long time before proceeding. A total of 2,932 trials (96.4%) remained for analysis.

We used linear mixed models (LMMs; Bates, Mächler, Bolker, & Walker, 2015) to analyze differences between response techniques and to evaluate possible effects of exposure duration to the virtual environments. The LMM approach allows to estimate by-subject and by-item variability simultaneously (e.g., by including a random intercept and/or slope for each participant and/or target object) and models individual participant responses without requiring prior aggregation, increasing power and robustness compared to traditional F1 versus F2 analysis of variance (Barr, Levy, Scheepers, & Tily, 2013). We computed a separate model for each dependent variable (Accuracy, Precision, Response Duration, and Subjective Workload). All models included the fixed factors response *technique* (object, cube, fixed, laser), *session* (1–4), and their interaction. The inclusion of the session factor accounted for possible effects of familiarization and exposure to the scenes over the course of the experiment. Models always included a random intercept term for participants (accounting for the fact that participants might differ in their overall baseline performance in the experiment). Additional random factors (random intercept per target object and random slopes for response technique or session) were added via forward model selection (Barr et al., 2013) wherever the likelihood ratio tests (LRTs) indicated a significant improvement in model fit. The final factor structure and number of observations for each model are reported together with the model results.

LMMs were fit using the *lme4* (Bates et al., 2015) and *lmerTest* (Kuznetsova, Brockhoff, & Christensen, 2017) R packages, and post hoc tests were computed using *emmeans*. All statistical effects were evaluated at an alpha level of α = .05, and the Holm method was used to correct for multiple comparisons where appropriate. The *p* values for LMMs were obtained using Satterthwaite's method to compute degrees of freedom, included in the *lmerTest* package. Data and code for this study are available at https://osf.io/qr5y9/.

Our sample size of 16 participants was chosen to allow an equal number of repetitions of each response technique in each of the four experimental blocks and was comparable to prior work that investigated endpoint errors for remembered object locations in VR (Klinghammer et al., 2016). We computed statistical power estimates for this sample size for the Hotelling Lawley Trace test within the GLIMMPSE online power analysis tool (Kreidler et al., 2013). To detect a main effect of endpoint *accuracy* between techniques (our main hypothesis), assuming a hypothetical endpoint mean difference of 0.01 m (standard deviation of 0.05 m) based on prior work (Klinghammer et al., 2016) yielded an estimated power of 0.922 at our chosen Type I error rate of 0.05. For *response duration*, assuming a 1-s difference (1-s *SD*) between techniques (roughly comparable to the short vs. unlimited pointer in Batmaz & Stuerzlinger, 2020) yielded a power asymptotically approaching 1.0.

## Results

### Spatial accuracy

**Figure 3.**
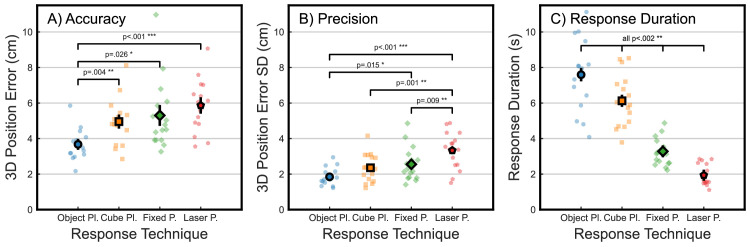
Spatial and temporal metrics for each of the four response techniques. (**A**) Accuracy, or average endpoint error (3D Euclidean distance between object and reported position). (**B**) Precision, or average endpoint error variability (standard deviations of 3D Euclidean distance between object and reported position). (**C**) Average response duration (time between the onset of the response phase and controller button press). Small markers indicate average individual participant data in all plots, and large markers display estimated marginal means from LMM analysis. Error bars show ± 1 *SEM*.

Participants were generally able to perform all four techniques easily and report the positions of previously seen objects in the larger scene with an accuracy of 10 cm or less, as shown in Figure 2 for the kitchen (A) and office scene (B). Individual spatial accuracy (defined as the 3D Euclidean distance between the true object position and the participant's reported endpoint) and LMM-estimated marginal means are presented in Figure 3A. The final model for accuracy was fit on all 2,932 valid observations (see Methods) and included random intercept coefficients for participant and target as well as a random slope coefficient for response technique (final model formula: *Positional* e*rror ∼ 1 + technique + session + technique:session + (1 + technique | participant) + (1 + technique | target)*). LMM analysis yielded a significant main effect of technique on object position errors (*F*(3, 16.6) = 13.3, *p* < 0.001). In post hoc analyses, the object placement technique was found to be significantly more accurate than all other methods (cube: *t*(15.7) = 4.1, *p* = 0.004, *d* = 0.53; fixed: *t*(16.1) = 3.1, *p* = 0.026, *d* = 0.67; laser: *t*(23.8) = 4.8, *p* < 0.001, *d* = 0.90). None of the other techniques differed significantly in accuracy during post hoc comparisons (all *t* < 1.7, all *p* > 0.3332). In contrast, there was no effect of session, indicating that participants did not become more accurate overall as the experiment progressed (*F*(3, 18.3) = 1.7, *p* = 0.200), nor was there a significant interaction between technique and session (*F*(9, 20.0) = 2.2, *p* = 0.063).

### Spatial endpoint bias

**Figure 4.**
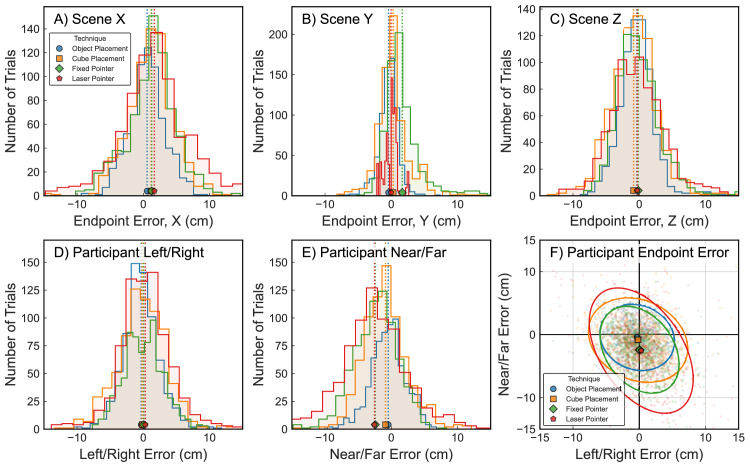
Positional endpoint error (individual response endpoints relative to the original object position) for each response technique. (**A**–**C**) Histograms of endpoint errors recorded along the Unity X-, Y-, and Z-axes, respectively (scene coordinates). (**D**, **E**) Histograms of endpoint error after assigning axes to match the participant's viewing direction in the scene (egocentric coordinates). D shows errors along the participant's subjective left–right axis (negative values indicate leftward error). (**E**) Along the subjective near versus far axis (negative values indicate undershooting the target). (**F**) Scatterplot of left–right and near–far egocentric endpoint errors (“bird's-eye view”). Colored markers in all plots and dotted lines in histograms denote mean endpoint positions, and ellipses in F show 95% confidence ellipses for each response technique. Plots based on all recorded endpoints (∼700 samples per technique).

Spatial accuracy as defined in the previous section only considers the distance between the participants’ endpoints and the target but does not provide information about possible directional bias. To determine if responses were directionally biased within the virtual scene (e.g., due to idiosyncrasies in the implementation of each response technique), Figure 4 displays histograms of signed endpoint errors along the Unity X, Y, and Z coordinate axes (scene coordinates, Figures 4A–C). Additionally, we were interested in whether participants’ responses were biased in relation to their own position and viewing direction, independent of where in the scene the target was located (egocentric coordinates). To this end, Figures 4D and 4E also plot histograms of individual endpoint errors but aligned to the participant's forward direction dependent on which side of the room the target was located. The result of this transformation are errors along an egocentric left–right axis (Figure 4D) as well as a near–far or undershoot–overshoot axis (Figure 4E). For better visualization, endpoints relative to the object in this “egocentric space” are also shown as a top-down scatterplot in Figure 4F.

To investigate directional bias, we performed Bonferroni-corrected one-sample *t*-tests against zero error for all coordinate axes and response techniques (3 scene axes + 2 egocentric axes × 4 techniques = 20 tests). Note that Shapiro–Wilk tests reported deviation from normality for all axes except one (X-axis/object placement: W = 0.996, *p* = 0.053; all other W > 0.084, *p* < 0.046). However, visual inspection of Q–Q plots and the histograms in Figure 4 indicated data close to a normal distribution, so we attributed this result to the large sample of valid trials included in this analysis (between 702 and 749 trials, depending on axis and technique) and chose to perform *t*-tests rather than select a nonparametric method.

In *scene coordinates* (Figures 4A–C), all four techniques showed a bias along the positive X-axis (mean error: object: 0.57 cm, cube: 1.18 cm, fixed: 1.26 cm, laser: 1.67 cm; all *t* > 5.41, all *p* < 0.001). For the Y-axis (endpoint height), this was also true for all techniques except the laser pointer (object: –0.42 cm, cube: 0.29 cm, fixed: 1.66 cm; all *t* > 3.22, all *p* < 0.001). For the laser technique, the absence of a significant Y-axis bias (laser mean: –0.03 cm; *t* = 0.88, *p* = 1.0) is expected, as this method determined its endpoint by intersecting the laser “ray” with object surfaces in the scene. Finally, along the scene Z-axis, both placement techniques showed a significant Z bias (object: –0.37 cm, cube: –0.86 cm; all *t* > 3.73, all *p* < 0.005), while both pointing methods did not (fixed: –0.15 cm, laser: –0.27 cm; all *t* < 2.0, all *p* > 0.937). It is of note that the largest average bias reported here (1.67 cm for the laser pointer in X direction) is still much smaller than the average absolute errors reported in Figure 3A (3.67 cm for object placement), making it unlikely that any overall accuracy results are driven by a bias along one specific coordinate axis.

An interesting pattern emerged when data were analyzed further in *egocentric coordinates* (Figures 4D–F). Participants’ responses were highly accurate in the egocentric left–right direction with no significant bias for any technique (mean error: object: –0.32 cm, cube: –0.18 cm, fixed: 0.04 cm, laser: 0.30 cm; all *t* < 3.0, all *p* > 0.056). At the same time, they undershot egocentric target distance using all methods (object: –0.45 cm, cube: –0.87 cm, fixed: –2.42 cm, laser: –2.54 cm; all *t* > 4.59, all *p* < 0.001), with an especially pronounced effect for the two pointing techniques (cf. Figure 4F).

### Spatial precision

We defined spatial precision as the average variability for each technique and participant, computed as the standard deviation of endpoints across all objects for a given technique. The resulting individual values and LMM results are plotted in Figure 3B. Since computing standard deviations across all responses yields only one value per participant and session, the resulting final LMM for precision was fit on 64 observations (16 participants × 4 sessions/response techniques) and included a random intercept for each participant (model formula: *Standard deviation ∼ 1 + technique + session + technique:session + (1 | participant)*). The model yielded a significant main effect of response technique, indicating that some techniques were more consistent in their spatial reports than others (*F*(3, 33.8) = 13.9, *p* < 0.001). In post hoc contrasts, the object placement technique was found to be more precise than the fixed pointer (*t*(33.2) = 3.0, *p* = 0.015, *d* = 1.07) and laser pointer (*t*(33.2) = 6.4, *p* < 0.001, *d* = 2.25) but not different from the cube placement technique (*t*(33.2) = 2.2, *p* = 0.072, *d* = 0.77). The laser pointing method was significantly less precise than the fixed pointing (*t*(33.2) = 3.3, *p* = 0.009, *d* = 1.18) and cube placement methods (*t*(33.2) = 4.2, *p* = 0.001, *d* = 1.47). Cube placement and fixed pointing achieved comparable precision (*t*(33.2) = 0.8, *p* = 0.410, *d* = 0.30). There was further no significant effect of session on precision (*F*(3, 33.8) = 2.3, *p* = 0.095) and no interaction between technique and session (F(9, 42.3) = 0.8, *p* = 0.654). Therefore, the placement conditions were comparable with one another in precision, followed by lower precision in the fixed and then laser pointing techniques.

### Response duration

To determine how quickly participants reported remembered object positions using each of the four techniques, we defined response duration as the time between the onset of the response phase (i.e., after the scene reappeared with the target object missing, independent of how much time participants had spent exploring the scene in the initial preview phase) and the time when participants confirmed the pointing action or object position using the trigger button. The resulting times are shown in Figure 3C, averaged across objects for each individual participant (small markers) and across participants as LMM-estimated marginal means (large markers, black outline). The LMM for response duration was fit using all 2,932 valid observations (see Methods). The final model included random intercepts for participant and target plus random slopes coefficients for session (per participant) and technique (per target; final formula: *Duration ∼ 1 + technique + session + technique:session + (1 + session | participant) + (1 + technique | target)*). This analysis showed a significant main effect of response technique (*F*(3, 26.5) = 97.4, *p* < 0.001). Additional post hoc analysis revealed significant differences for all pairwise comparisons (all *t*(>19.4) > 3.6, all *p* < 0.002, all *d* ≥ 1.00), indicating that the four response techniques differed in response duration. There was no significant main effect of session (*F*(3, 10.6) = 0.5, *p* = 0.704), meaning that average response durations did not change over the course of the experiment. Lastly, there was no interaction between technique and session (*F*(9, 18.0) = 1.2, *p* = 0.379).

### Object interactions

**Figure 5.**
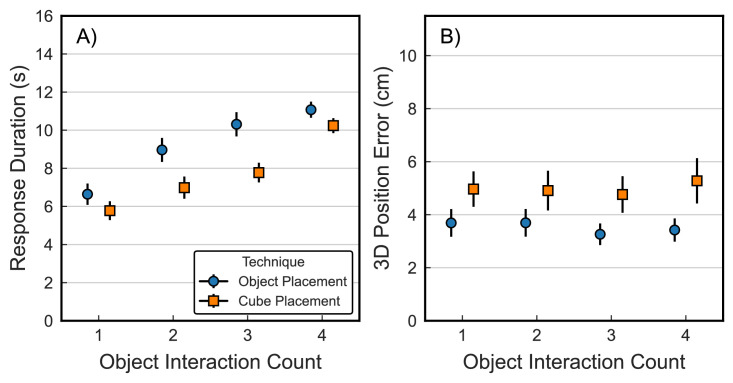
Average response duration (left) and average 3D object position error at the end of a trial, shown as a function of number of object interactions (grab and release) in each trial. Dotted lines show across-participant average from Figures 3A and 3C, respectively. Error bars show ± 1 *SEM*. Only interaction counts that appeared in at least 10 trials are shown.

The pointing techniques (fixed and laser) only allowed for a single endpoint response upon pressing the trigger button, which immediately ended the trial. In contrast, the placement techniques (object and cube) allowed for multiple interactions with the target object by grabbing and releasing it multiple times (e.g., to fine-tune its position and orientation prior to trial termination). As such, we were interested in exploring the number of times participants interacted with the object during these two tasks and whether this was related to response duration and final spatial accuracy at the end of a trial. In the majority of trials, the target object was grabbed and released only once (object: 72.9%, cube: 77.0%), although two (object: 18.1%, cube: 15.7%) and three (object: 6.3%, cube: 5.3%) interactions were also fairly common. The maximum were seven interactions, which appeared in exactly one trial. Figure 5 plots average response durations (A) and average final position error (B), averaged across all trials that received between one and four separate interaction events (meaning that, for example, no data are shown for five interactions because fewer than 10 trials existed in which participants interacted with the object exactly five times).

Additional object interactions demonstrate a noticeable increase in response durations for both the object and cube placement techniques. However, repositioning an object multiple times in this way did *not* lead to lower final position error and merely allowed participants to achieve similar final accuracies, regardless of the accuracy of the first placement.

### Object orientation

**Figure 6.**
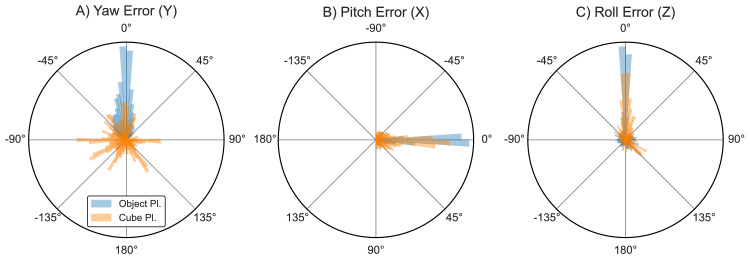
Polar histogram of orientation error (deviation from original object's forward vector) for the object placement (blue) and cube placement techniques (orange). Yaw (Y) error represents a left (negative values) or right (positive values) deviation around the vertical axis, while pitch (X) and roll (Z) indicate deviations from an object's original alignment to its resting surface. Histogram bin width 4° (90 bins total). Pitch error plot rotated to better visualize “up” (negative) versus “down” (positive) error.

Besides positional accuracy and precision, the two placement techniques (*object* and *cube*) further enabled us to include an exploratory analysis of how participants *oriented* the response object in 3D space as part of their response (i.e., whether they rotated the target object or cube to match the remembered object's orientation). Figure 6 plots angular histograms of the yaw (Y), pitch (X), and roll (Z) errors recorded during only object and cube placement.

In the object placement technique (blue), errors strongly cluster around the original orientation across all three axes, as evidenced by small mean error and standard deviations (average signed errors: *yaw* mean: 5.1°, *SD* 16.1°; *pitch* mean: –0.8°, *SD* 6.0°; *roll* mean: 2.7°, *SD* 43.0°), suggesting that participants made great effort to reproduce the original object orientation and align the object with the surface the target rested on, despite not being explicitly instructed to do so. In the cube placement technique, errors were more variable, especially for yaw and roll (*yaw* mean: 23.0°, *SD* 96.7°; *pitch* mean: –2.4°, *SD* 20.5°; *roll* mean: 19.1°, *SD* 52.4°), with errors clustering around specific orientations (Figure 6A; orange). This suggests that participants tried to align one side of the cube to the resting surface (note that all sides of the cube showed the same uniform green texture, meaning that there was no obvious “forward” direction when placing the cube).

### Subjective workload

**Figure 7.**
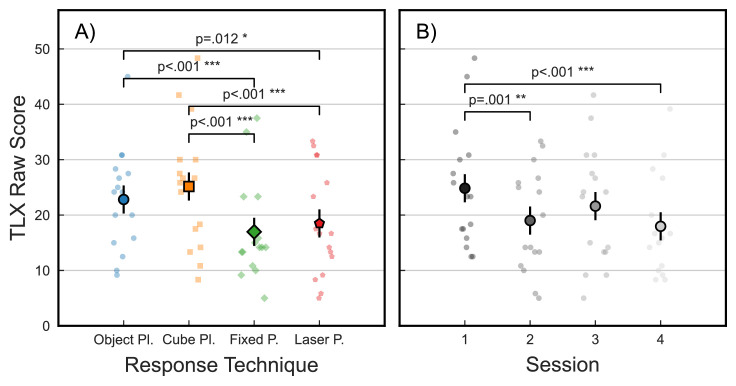
Subjective workload (raw NASA TLX scores) for each participant (small markers) and LMM estimated marginal means across participants (large markers), plotted by response technique (left) and experimental session (right). Error bars indicate ± 1 *SEM*.

Finally, in addition to the spatial and temporal considerations used as objective measures of participants’ performance, we used the NASA TLX questionnaire to assess their subjective workload in performing the four response techniques. Figure 7 plots raw TLX summary scores for each participant (small markers) and corresponding estimated marginal means from LMM analysis (large markers, black outline), separately by response technique (left) and experimental session (right). Because each participant filled out the TLX questionnaire after each session (technique), the linear mixed model was fit on 64 observations (16 participants × 4 sessions) and includes a random intercept coefficient for participant (resulting formula: *TLX ∼ 1 + technique + session + technique:session + (1 | participant)*).

Statistical analysis revealed a significant main effect of response technique (*F*(3, 32.9) = 14.4, *p* < 0.001, cf. Figure 7A). Post hoc analysis showed that this effect was mainly driven by differences between the placement and pointing techniques (cube—laser: *t*(33.0) = 4.7, *p* < 0.001, *d* = 1.67; cube—fixed: *t*(33.0) = 5.8, *p* < 0.001, *d* = 2.05; object—laser: *t*(33.0) = 3.1, *p* = 0.012, *d* = 1.09; object—fixed: *t*(33.0) = 4.1, *p* < 0.001, *d* = 1.47), while individual pointing and placement techniques did not differ within each category (object—cube: *t*(33.0) = 1.7, *p* = 0.211, *d* = 0.59; laser—fixed: *t*(33.0) = 1.1, *p* = 0.291; *d* = 0.38). Additionally, subjective workload also significantly decreased over the four experimental sessions, as participants became familiar with the virtual environment and task (*F*(3, 32.9) = 9.5, *p* < 0.001, cf. Figure 7B). Significant post hoc results were found for Session 1 compared to Session 2 (*t*(33.0) = 4.1, *p* = 0.0011, *d* = 1.47) and Session 4 (*t*(33.0) = 4.9, *p* < 0.001, *d* = 1.73), suggesting that initial high workload in the first session could mainly drive this effect. No other pairwise comparisons were significant (all *t* < 2.6, all *p* > 0.057, all *d* < 0.92). Lastly, no interaction between response technique and session was found for subjective workload (*F*(9, 34.7) = 1.1, *p* = 0.360).

## Discussion

In the present study, we evaluated four different techniques to indicate the remembered position of a previously seen object in a naturalistic VR scene, two placement and two pointing techniques. We anticipated that object placement would yield the highest accuracy, while the laser pointer would be the fastest method due to the fact that it required the least amount of movement. We found that placing the target object was indeed the most accurate method but also the slowest by a factor of 2–4 compared to the other techniques. Participants adjusted object position multiple times and tried to match the angular orientation of the object (to no additional gain in positional accuracy). Pointing methods outperformed the two placement methods in interaction speed, with pointing onto a resting surface using the virtual laser pointer being the fastest method. Participants’ perceived workload was overall lower for pointing compared to placement techniques.

A main goal of this study was to test whether the human behavior reported for online visuomotor actions also transfers to memory-guided actions in VR. To foster naturalistic behavior, we allowed natural walking in our VR scene and avoided constraining our participants’ behavior by not giving specific instructions about how they should behave (e.g., how to define accuracy, the number of times the object could/should be maneuvered, whether they should walk or remain at the start position, etc.). With these unconstrained instructions, we found that object placement was significantly more accurate than all other methods, including placing a constant reference cube that required identical use of the controller. The remaining response techniques (cube, fixed, and laser pointer) all achieved comparable accuracy. Based on these results, arranging virtual objects using the controller turned out to be an optimal interaction choice in prior work measuring semantic scene relationships and memory performance (Draschkow & Võ, 2017; Helbing et al., 2020), maximizing endpoint accuracy. Our study exclusively used the VR controller to collect responses, and therefore, we cannot directly draw comparisons with prior work that used direct index finger pointing (Karimpur, Eftekharifar, et al., 2020; Klinghammer et al., 2016). Pointing using a virtual cursor moved by the participant's tracked index finger tip might be argued to resemble the fixed, short pointer of our task; however, finger pointing and use of a tool such as the controller are likely to engage different mechanisms for action. Similarly, a direct comparison of endpoint accuracy and motor behavior when placing a real versus a virtual object (e.g., Karimpur, Kurz, et al., 2020) would be an exciting future research opportunity as augmented reality devices become more widely available to blend real and virtual environments in sensorimotor research (Carmigniani et al., 2011).

Analyzing directional endpoint bias along the three coordinate axes that spanned the virtual scene uncovered small but significant bias in the positive X and negative Z directions (i.e., in the horizontal plane within the scene), as well as small biases in different directions along the Y (vertical) axis. Since the scene coordinate system was aligned with the real-world lab, systematic error could indicate measurement bias resulting from the SteamVR positional tracking system (Niehorster, Li, & Lappe, 2017). However, since biases were much smaller than the smallest average error reported in the object placement task, it is unlikely that such a bias would have had a strong impact on our comparison of response techniques, especially given the within-subject design employed. The specific biases seen in scene coordinates might also be an artifact of participant-relative, egocentric bias across different viewing directions: Participants responded accurately in the horizontal direction but consistently undershot the actual target position using all methods. Undershoots of distance in VR have been consistently reported before (Renner, Velichkovsky, & Helmert, 2013), although it is of note that the undershoot bias was larger for pointing compared to placement techniques. While an angular downward bias of the pointer ray might be an explanation for the laser pointer technique, which was almost always used from a long distance away, this cannot explain the comparable bias seen in the fixed pointing task. It is more likely that the difference in spatial cues available in each technique allows for a more correct estimation of distance when performing a placement task.

The higher spatial accuracy we found for object placement compared to all other techniques might be explained by the availability of additional cues related to the target object, such as 3D shape, extent, and center of mass, which would allow for more accurate positioning relative to the surrounding objects and environment. Previous studies suggest that surrounding landmarks are used to localize objects (Fiehler, Wolf, Klinghammer, & Blohm, 2014; Klinghammer et al., 2016) and can improve spatial accuracy and precision in memory-guided actions (Hay & Redon, 2006; Obhi & Goodale, 2005; Schütz, Henriques, & Fiehler, 2013; Sheth & Shimojo, 2004). Therefore, availability of these cues might be a required consideration for experiments studying memory-guided actions. It may also be the case that additional mechanisms, such as “magnetic” snapping to surfaces (Kitamura, Ogata, & Kishino, 2002), can help to further reduce overall object positioning errors in practice. Here, we chose not to employ any such “assistive” mechanics or even simulated physics (e.g., objects dropping onto resting surfaces), because we aimed to give participants full control over the placeable target object in order to best report their spatial memory of the target. Future work could investigate whether adding gravity, for example, increases accuracy due to higher realism or possibly even reduces accuracy due to imposing extra movement onto the object (e.g., dropping or tumbling). Regardless of these factors, object placement appears to be the best technique for a researcher looking to optimize for response accuracy.

Besides position, participants also tried to match the original orientation of the target object, despite receiving no specific instructions to do so. Notably, the present study did not explicitly randomize object orientation for each response phase and therefore cannot answer whether this was an explicit strategy or, if so, whether participants aligned to an object's remembered orientation or instead to visible cues such as surfaces and walls. So far, no research on spatial coding for action has considered the use of landmarks in reproducing angular orientations, making this an interesting opportunity for future work.

For spatial precision, defined as the variability between different endpoints reported using the same technique, we found object placement to be the most precise technique, while the laser pointer showed significantly higher variability than any other method. This is consistent with intuition, as pointing errors using a raycast are known to scale with distance due to the amplification of even small angular variability or jitter (Argelaguet & Andujar, 2013; Batmaz, Seraji, Kneifel, & Stuerzlinger, 2020; Poupyrev, Ichikawa, Weghorst, & Billinghurst, 1998). Notably, all but one participant stayed near the center of the virtual room while reporting positions using the laser pointer, which also contributed to the much lower response durations for this method. Accuracy and precision in the long-range laser pointing technique may therefore have also been affected by the proximity-/task-related consequences associated with being within or outside of peri-personal (reachable) space (Gamberini, Carlesso, Seraglia, & Craighero, 2013). The walking component associated with all methods except the laser may provide additional cues to assist natural sensorimotor processes that are absent when standing and indicating a remembered object position from afar (Apfelbaum, Pelah, & Peli, 2007).

It is of note that endpoint accuracy and precision as measured using all of the tested response techniques by definition represent a combination of different sources of uncertainty, such as variability in the actual spatial memory that participants are asked to reproduce, but also motor variability introduced by each specific interaction technique. We aimed to standardize the encoding phase of the task as much as possible across response techniques, for example, by requiring participants to start each trial from the same central position and facing a predefined direction in the scene. However, because response techniques were consistent within a session, it is conceivable that participants utilized different cues during encoding to prepare for an upcoming response, such as encoding an egocentric direction vector for a laser pointer response while relying more on the position of landmark objects for an upcoming object placement response. The current experimental design does not offer a simple way to separate memory and motor variability, but future work could measure the memory representation directly while avoiding motor variability, for example, by asking participants to detect a perceptual difference between two sequential, slightly offset object presentations in a psychophysics paradigm. Such estimates could then be compared to the variability measured using pointing or placement to determine the relevant variability components. Due to the fact that response techniques have rarely been compared directly, it remains unclear whether the choice of response method might have affected encoding strategies in recent spatial memory studies (e.g., Helbing et al., 2020), but any within-subjects comparisons using the same technique would remain valid.

Besides response accuracy and precision, the duration of the experiment can also play an important role when deciding about the number of experimental conditions, for example, to avoid participant fatigue or be able to test specific groups such as older adults or patients. We observed that the four tested response techniques varied strongly in the time it took participants to complete a trial, ranging from about 7.5 s for object placement to below 2 s for the laser pointer. This was also visible when considering the average durations of each session, which ranged from 9.3 min for the laser pointer condition to 15.3 min for the object placement task (cube: 14.0 min, fixed: 11.9 min). In the case of the virtual object placement method, the additional time was associated with a higher number of object manipulations (grab and release), which extended response durations but did not appear to improve final position accuracy (note that due to the way we recorded movement data, it was not possible to analyze whether repeated manipulations helped improve *orientation* accuracy in this study). The fact that the laser pointer required no movement around the scene and that participants took advantage of this, as shown by recorded headset movement data, contributed to its great advantage in response times, albeit at the expense of accuracy and precision. Therefore, if a large number of trials are required or if responding quickly to events in a virtual experience is necessary, the virtual laser pointer can be a desirable technique for visuomotor tasks. The same is true if the stimulus material can tolerate larger errors than seen for placement or if the greater number of trials can make up for reduced precision. Finally, this method still allows for sensorimotor research with limited lab space (i.e., pointing from a seated position).

After each session, we presented participants with the NASA TLX questionnaire to measure their subjective impression of effort and workload associated with performing each technique. Pointing techniques (laser and fixed) received generally lower scores than the placement tasks (object and cube). This finding is in agreement with qualitative statements by participants about the laser being “faster” and “easier,” but it is notable that the fixed pointer was rated similarly to the laser pointer despite the necessity to walk close to the target object's prior location. This suggests that the higher perceived workload in the placement conditions might be more related to the active (and potentially multiple) “grab” interactions necessary to place the target object. As mentioned previously, repeated object repositioning did not lead to higher final spatial accuracy, but it may have related to a desire to get the object “exactly right” (as described by one participant) or correct an imperfect first attempt at positioning and thus achieve comparable accuracy for all trials within a session. Instructing or enforcing a single grab interaction per trial might reduce trial durations and subjective workload but could potentially also reduce final endpoint accuracy due to removing the possibility to correct initial mistakes. We encouraged participants to give general verbal feedback about the study after their last session and also asked them about their preferred position reporting technique. The latter question uncovered two main clusters of participants: The majority preferred the object placement task, generally citing that it felt more “natural,” “engaging,” or “fun”; however, a minority preferred the laser pointer for its efficiency (“faster,” “I did not have to walk with the laser pointer”). We did not explicitly instruct participants to stay in the center of the virtual room after the beginning of the response phase; however, almost everyone pointed from the center when using the laser, and only one participant walked closer to the object's prior position “to be more accurate.” Finally, two participants referred to the cube task as “the most frustrating” task. Taken together, balancing engagement with effort may serve to help in optimizing accuracy and response times for visuomotor tasks in virtual settings.

When considering all metrics in combination, the fixed pointer method represents a good middle ground between accuracy and interaction speed and is therefore a good default choice for user studies that investigate remembered 3D locations. Using a fixed pointer was previously reported to be slower than an unlimited raycast, although for a task in which participants did not have to physically move themselves in either case (Batmaz & Stuerzlinger, 2020). In contrast, our fixed and laser pointing conditions differed in that participants were required to walk to the remembered object position when using the fixed pointer. However, the significant difference in efficiency we found comparing walking (fixed) and standing (laser) was still much smaller than the difference of either method to any of the placement interactions. This suggests that interaction complexity, rather than the requirement to walk a few steps in VR, may be the main driver behind differences in response efficiency.

To sum up our results and make it easier for researchers to select an interaction method for a future study, Table 1 provides a high-level summary of participants’ average performance (estimated marginal means) across the different metrics for each response technique. Additionally, we have labeled the “best” and “worst” results for each metric (column) to facilitate comparisons between techniques and included subjective recommendations by the authors.

**Table 1.** Summary of participants’ behavioral metrics and subjective experience for the four response techniques to report a remembered object location in VR. *Notes*: NA = not available.

	Spatial accuracy	Spatial precision	Rotational accuracy	Response duration	Subjective workload	Recommendation
Object placement	3.67 cm highest	1.85 cm highest	5.1° highest	7.59 s slowest	22.8	Best for high accuracy or realism; slowest method
Cube placement	4.96 cm	2.36 cm	23.0° lowest	6.12 s	25.2 highest	No clear benefit over object placement; angular ambiguity
Fixed pointer	5.30 cm	2.55 cm	NA	3.28 s	17.0 lowest	Good middle ground between accuracy and response speed
Laser pointer	5.86 cm lowest	3.33 cm lowest	NA	1.94 s fastest	18.5	Best for fast responses or limited movement space

For the purposes of this study, we chose to use the center of an object's bottom contact surface as our definition of object position. Because the endpoint errors we found were relatively small and participants also tended to phrase objects as positioned “on” surfaces when speaking about their experience during the study, this definition appears to have been a reasonable choice for the tested research question. Other definitions of “position” in 3D space are possible, such as an object's center of mass or the center of its bounding box. The specific definition that an observer uses very likely depends on the task performed, such as when pointing to an object to disambiguate it from others versus pointing to an object's former position on a shelf. Comparing different definitions of object position and their impact on complex interactive tasks in VR is another promising area for future research.

In conclusion, we have evaluated four different response techniques to interactively report remembered object locations in a virtual environment and provide baseline metrics that can assist researchers in selecting an appropriate response technique for a future VR study, whether their goal is to achieve the highest accuracy and/or realism (object placement), a rapid collection of responses or make use of limited lab space (laser pointer), or a balanced default interaction (fixed pointer).
